# Insight into the Mechanisms of Low Coverage Adsorption of N-Alcohols on Single Walled Carbon Nanohorn

**DOI:** 10.3390/ma14144001

**Published:** 2021-07-17

**Authors:** Grzegorz Stanisław Szymański, Anna Kaczmarek-Kędziera, Monika Zięba, Piotr Kowalczyk, Artur Piotr Terzyk

**Affiliations:** 1 Physicochemistry of Carbon Materials Research Group, Faculty of Chemistry, Nicolaus Copernicus University, Gagarin Street 7, 87-100 Toruń, Poland; greg_ss@uni.torun.pl (G.S.S.); monikaventabal@gmail.com (M.Z.); 2 Faculty of Chemistry, Nicolaus Copernicus University, Gagarin Street 7, 87-100 Toruń, Poland; teoadk@umk.pl; 3 College of Science, Health, Engineering and Education, School of Engineering and Information Technology, Murdoch University, Perth, WA 6150, Australia; kowalczyk.piotr@wp.pl

**Keywords:** adsorption, carbon nanohorn, nanomaterials, chromatography, DFT

## Abstract

We report for the first time the chromatographic study of n-alcohols (from methanol to butanol) adsorption on single walled carbon nanohorn (SWCNH). Using measured temperature dependence of adsorption isotherms (373–433 K) the isosteric adsorption enthalpy is calculated and compared with the data reported for a graphite surface. It is concluded that a graphite surface is more homogeneous, and the enthalpy of adsorption on SWCNHs at zero coverage correlates well with molecular diameter and polarizability, suggesting leading role of dispersive interactions, i.e., no heteroatoms presence in the walls of SWCNH structures. Next using modern DFT approach we calculate the energy of n-alcohols interactions with a graphene sheet and with a single nanocone finally proposing a more realistic—double nanocone model. Obtained results suggest alcohols entrapping between SWCNH with OH groups located toward nanocones ends, leading to the conclusions about very promising future applications of SWCNHs in catalytic reactions with participation of n-alcohols.

## 1. Introduction

Single Walled Carbon Nanohorn (SWCNH) is widely applied as adsorbent and catalyst. The space available inside tubes forming SWCNH aggregates makes it possible to introduce different host molecules inside and, in this way, allowing the application of SWCNH as nanocontainers. Therefore, it was stipulated that SWCNH and other carbon nanoforms may find applications in hydrogen storage. Recent GCMC simulation results show that with increasing number of pentagons and decreasing apex angle, hydrogen storage inside SWCNH increases [1]. However, it is well known that due to low potential energy of carbon—hydrogen interactions, carbon adsorbents are inapplicable for efficient hydrogen storage [2]. Thus, metal doping is necessary to increase the potential energy of adsorbate—adsorbent interactions and finally, hydrogen storage. Banerjee et al. [3] studied theoretically the influence of SWCNH metal doping on hydrogen adsorption the authors showed that Li doping is the most promising. In fact, recently Sano et al. [4] proved that by a doping of SWCNH with metal alloys hydrogen storage can be remarkably increased. It is also important that Pt—supported SWCNH are expected to be efficient hydrogen storage materials after spillover reactions [5,6,7].

To improve adsorption properties of SWCNH aggregates some attempts were made to decrease pore size by subjecting the material to compression [8]. As for unmodified nanohorns the compression does not change the structure, remarkable changes are observed for defected SWCNHs (oxygenated and H_2_O_2_ treated) and they are induced by the mechanism of, so called, defect—migrated compression. The resultant microporous material shows relatively good methane adsorption properties. Methane enhanced capacity can be also obtained by adding of small amount of dispersed lanthanides [9]. This is due to the charge transfer effect observed only for SWCNH and not observed for other nanocarbons.

Summing up, considering SWCNH as adsorbent hydrogen and methane storage are the most often reported, and adsorption data of other gases on SWCNHs are rarely met in literature. Among them Ohba et al. [10] used SF_6_ adsorption for the systematic characterisation of nanopores in SWCNH. Due to sieving effect nitrogen is able to penetrate the whole pore space while SF_6_ can be useful for nanoporosity characterisation. The Density Functional Theory (DFT) analysis of defects created by the presence of surface oxygen groups on SWCNH was discussed by De Souza et al. [11]. Krungleviciute et al. [12] studied adsorption of Ne and CF_4_ on closed SWCNH at two temperatures. The SSA determined by the B-point method was temperature dependent and ranged between 298 and 393 m^2^/g. Authors concluded that the previous studies on single walled carbon nanotubes did not reveal the differences in SSA for different adsorbates. In contrast, in the case of adsorption between SWCNH, the wedge-like shape of pores formed between tubes provides differences. Following this interesting observation in the current study we need to check the effect of SWCNH walls curvature on adsorption of n-alcohols. We also need to check the homogeneity of SWCNH walls and exclude the possible presence of polar heteroatoms. This is interesting topic for future application of SWCNH in catalysis, because wide application of carbon materials in (for example) alcohols dehydration catalytic processes [13]. Alcohol’s adsorption is also very important for application of carbon nanomaterials in fuel cells [14] and supercapacitors [15]. Obtained results will be compared with the data reported for n-alcohols adsorbed on homogeneous graphite surface.

## 2. Materials and Methods

### 2.1. Adsorbent and Adsorbates

**Figure 1:**
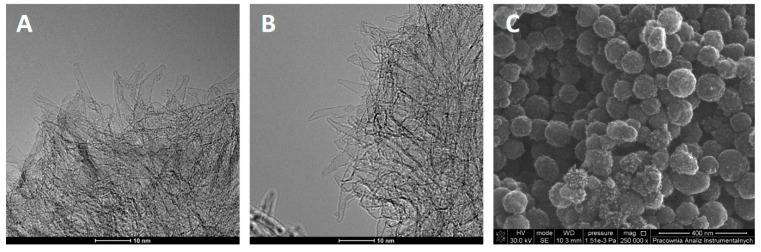
HRTEM (**A**,**B**) and SEM (**C**) images of studied SWCNH.

SWCNHs were purchased from Nec Company (Tokyo, Japan). *n*-Alcohols C_1_-C_4_ (A.R. grade) were used as adsorbates (they are denoted as MeOH (methanol), EtOH (ethanol), PrOH (n-propanol) and BuOH (n-butanol). Figure 1 shows the morphology of SWCNH obtained using high-resolution transmission electron microscopy (HRTEM, Tecnai G2 F20 X-Twin, FEI Europe, Eindhoven, North Brabant, Netherlands). Measurement was carried out with an accelerating voltage of 200 kV. The Scanning electron microscopy (SEM) measurements were performed using Quantax 200 with XFlash 4010 detector (Bruker AXS machine). To perform the High Resolution Transmission Electron Microscopy (HRTEM) measurements SWCNHs were dispersed in ethanol (96%, pure p.a., Chempur, Piekary, Śląskie, Poland) in concentration of 0.001 mg·mL using sonication (30 s, 18 W, Sonoplus Mini20, Bandelin, Berlin, Germany). Then the TEM grid (Lacey Carbon film on copper TEM grid, mesh 400, PIK Instruments, Piaseczno, Poland) was immersed in the SWCNHs dispersion and air-dried at room temperature. Images were obtained using a Transmission Electron Microscope (FEI, Tecnai F20 S-Twin, Particulate Systems, Norcross, GA, USA) operated at 200 kV.

### 2.2. Chromatographic Measurements

The chromatographic measurements were performed using a Chrom 4 gas chromatograph (Laboratorni Pristroje, Prague, Czech Republic) equiped with a computer to process the data. A flame ionization detector (FID) was used. A glass column (30 cm × 2 mm I.D.) with an absorbent bed length of 22 cm corresponding to 0.12 g of CNHs was used. The samples were packed by a gentle tapping and fixed with glass wool at the top and bottom of the sample. Silane-treated glass column and wool were used to avoid an alcohol adsorption on glass walls of the column.

Before experiments, the column containing SWCNHs was conditioned at 433 K for 3 h under a flow of helium (30 mL/min). In order to avoid detector contamination, the outlet of the column was not connected to the detector during this period.

Alcohols (0.5 µL) were injected into the column using a Hamilton microsyringe, and the temperature of the injection device was equal to393 K. Adsorption isotherms were measured in the range of 373–433 K in 10 K steps at carrier gas flowrates 40 min^−1^. At each temperature the sample was thermostated for 1 h. This flow rate of a carrier gas was measured using the detector outlet with a soap bubble flowmeter and was corrected for pressure drop in the column using James–Martin gas compressibility factor, correction at column temperature was also made [16].

Adsorption isotherms were determined using the well—known peak profile method [16,17]. The peaks obtained were unsymmetrical for all the adsorption systems. It means that the diffusion has strong influence on the rate of adsorption equilibrium establishment. This is why during calculation of adsorption isotherms the diffusion effect was eliminated using the postulates of Dollimore et al. [18].

Form obtained isotherms we calculated the isosteric heat of adsorption (*q_ist_*). It was carried out by converting adsorption data, i.e., using isosteres—the plots of partial pressures (ln *p*) versus temperatures (1/*T*) at constant adsorption. Next the Clausius-Clapeyron equation [16,19] was used in the form:

(1) $$q_{ist}=R{\left[\frac{\partial lnp}{\partial \left(\frac{1}{T}\right)}\right]}_{a}$$

where *R* is the gas constant, and *a* is adsorption.

### 2.3. DFT Calculations

To support the results of our study the DFT calculations were applied. Three sets of data have been obtained: the equilibrium configurations and interaction energies of alcohols adsorbed on graphene, on SWCNH wall and between two SWCNH. SWCNH with the apex angle 19.2° (i.e., close to experimental one) was modelled using Nanotube Modeller ver. 1.8.0 (JCrystalSoft, http://www.jcrystal.com/) software as previously [20].

Full geometry optimization for ground singlet state in vacuum has been performed for the investigated systems within the ωB97X-D/6-31G(d) approach. The selected range-separated functional containing the 100% of exact exchange in the long-range limit and the a posteriori dispersion correction has been proven to perform well for the weak intermolecular interactions and, among other, dispersion-bound aromatic complexes [21,22]. Applied graphene model consists of one sheet of 38 carbon atoms and the nanocone is built of 50 carbon atoms. Double cones are constructed of two identical cones (all together 100 carbon atoms), connected with the two C–C bonds forming the hexagon between the two structures. The partial geometry optimization for double cones has been performed with the frozen distance between the cone apexes equal to 6.80 Å.

In all types of systems, the edge carbon atoms are saturated with hydrogens. The nature of the stationary points for the fully optimized complexes of graphene and single cone has been confirmed by the harmonic vibration analysis. Due to the system size for the double cone complexes, the supermolecular approach has been applied for the estimation of the material-alcohol interaction energy with the ωB97X-D functional and Pople basis set containing a set of diffuse function on the heavy atoms, namely 6-31+G(d), in order to provide the reliable quality-to-cost ratio. Additionally, for the graphene sheet and single cone complexes, energy decomposition within the Symmetry—Adapted Perturbation Theory (SAPT0) procedure with the aug-cc-pVDZ Dunning correlation consistent basis set augmented with the set of diffuse functions has been carried out. Such a combination of the SAPT expansion limitation (namely the zero-th order with respect to the intramonomer correlation) with the moderate basis set is expected to guarantee good performance, ensuring the qualitative agreement of the obtained data with the benchmark values due to the optimal error cancellation [23].

The total interaction energy estimated within the SAPT0 approach can be decomposed into the following contributions with the clear physically derived origin:

*E *_SAPT0_* = E_elst_ + E_exch_ + E_ind,r_ + E_exch−ind,r_ + E_disp_ + E_exch−disp_* (2)

where the definitions of the subsequent terms can be found in the original works by Jeziorski, Moszynski and Szalewicz and in the review by Hohenstein and Sherrill [24,25,26], and the *E_elst_* denotes the energy of electrostatic, *E_exch_*—energy of exchange, *E_ind,r_ + E_exch−ind,r_* energy of induction and *E_disp_ + E_exch−disp_* energy of dispersion interactions, respectively.

## 3. Results and Discussion

Collected in Figure 1A and B HRTEM images confirm that studied SWCNH form so called dahlia structures, with tubes of 2–5 nm in diameter. SEM images (a typical one is shown in Figure 1C) reveal that the aggregates are of 50–100 nm wide.

**Figure 2:**
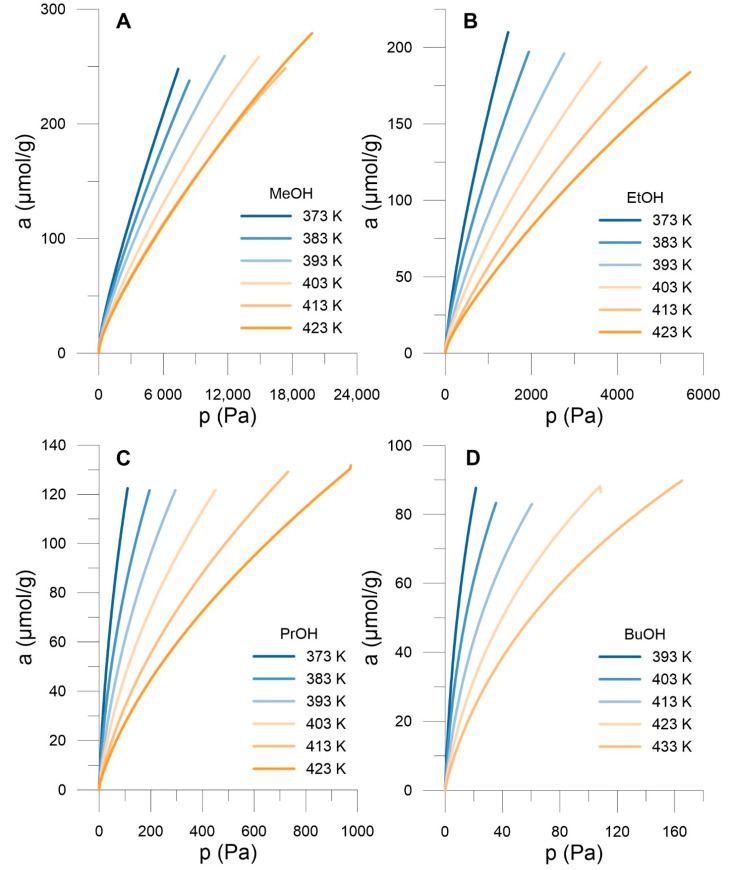
Adsorption isotherms of n-alcohols on SWCNH in the range of 373–433 K, MeOH (**A**), Et OH (**B**), PrOH (**C**) and BuOH (**D**).

**Figure 3:**
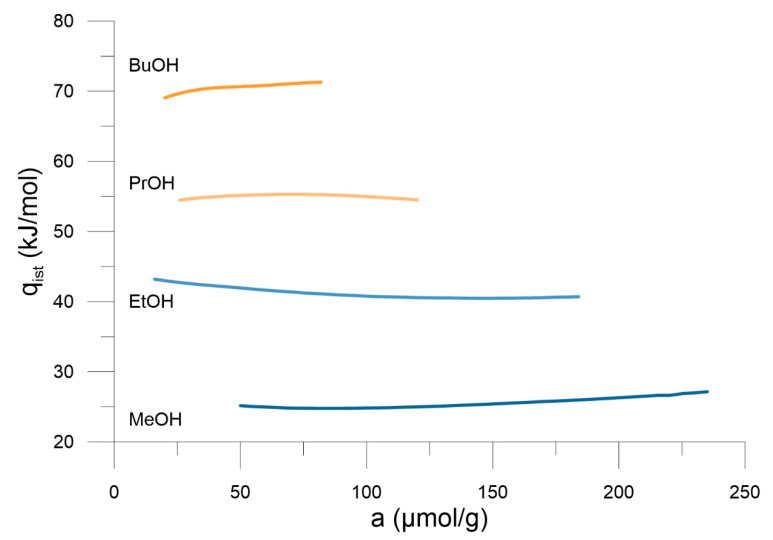
The plots of isosteric enthalpy of n-alcohols adsorption on SWCNH in the range of 373–433 K.

Figure 2 presents obtained adsorption isotherms. One can observe the decrease in adsorption with the rise in temperature, and at a given temperature the rise in adsorption with increasing alcohol chain length (Figure 2A–D). The exothermic nature of the process leads to the negative isosteric enthalpy of adsorption (Figure 3), increasing with the alcohol chain length. Since we are at low coverages, the process is dominated by adsorbate—adsorbent interactions and this is why adsorption increases with the rise in isosteric adsorption enthalpy (i.e., solid—fluid potential energy of interactions).

To determine the values of adsorption enthalpy at zero coverage we applied the same procedure as proposed previously [27].

Taking the obtained data, we tried to check the homogeneity of SWCNH walls. To do this the relation proposed by Berezin and Buryak [28,29] was applied. The authors showed that for the perfect homogeneous surface the following relation between the isosteric enthalpy of adsorption at zero coverage (Δ*U*_0_) and the critical parameters of adsorbed molecules *T_c_* and *p_c_* (i.e., the critical temperature and pressure, respectively) occurs:

(3) $$-\Delta U_{0}=DT_{C}/{\left(p_{c}\right)}^{1/2}$$

Equation (3) was developed for a perfect homogeneous carbon black surface, thus it can be applied for checking the homogeneity of a studied SWCNHs. Figure 3 shows the plot of Equation (2) for the data of n-alcohols adsorption on graphite [30,31] and on SWCNH.

**Figure 4:**
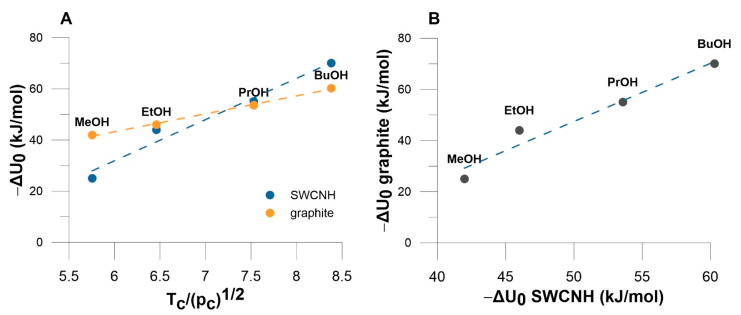
(**A**) The plot of isosteric enthalpy at zero coverage on graphite and on SWCNH in the coordinates of Equation (3). The values of determination coefficients (DC) are equal to 0.9963 and 0.9693 for correlation on graphite and SWCNH, respectively. (**B**) The comparison of enthalpy on zero coverage on graphite and SWCNH (DC = 0.9527).

The comparison of the values collected in Figure 4 leads to the conclusion about smaller energy of MeOH adsorption on SWCNH than on graphite. In contrast, larger energy on SWCNH is observed for BuOH (Figure 4B). For two remaining alcohols the energy of interactions on SWCNH and graphene are similar. This leads to the conclusion that based on the experimental data collected on Figure 4, graphite surface is slightly more homogeneous than SWCNH. This can be caused by the differences in the nature of adsorption space. In fact, graphite can be called as flat-surfaced while in the case of SWCNHs the adsorption space is created between nanocones (see below). However, the effect of a cone curvature is visible for MeOH adsorption as decreasing adsorbent—adsorbate interaction energy, and in contrast—for BuOH, potential energy of solid-fluid interactions increased (see below).

**Figure 5:**
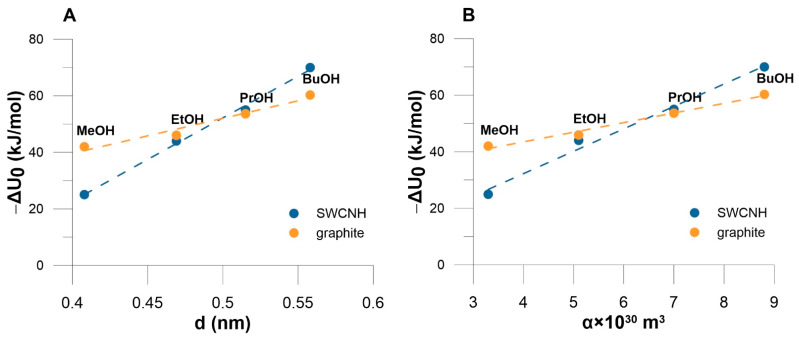
(**A**) The correlation between the enthalpy of adsorption at zero coverage and molecular diameter for adsorption on graphite and on SWCNH (DC = 0.9601 and 0.9963). (**B**) The correlation between the enthalpy of adsorption at zero coverage and molecular polarizability for adsorption on graphite and on SWCNH (DC = 0.9872 and 0.9877).

Figure 5 shows the relation between Δ*U*_0_, molecular diameter and polarizability of adsorbates. For studied alcohols, a good correlation is observed between polarizability and the molecular diameter both parameters correlate well with Δ*U*_0_. This type of correlation is a proof of dominant dispersion interactions in the studied systems.

**Figure 6:**
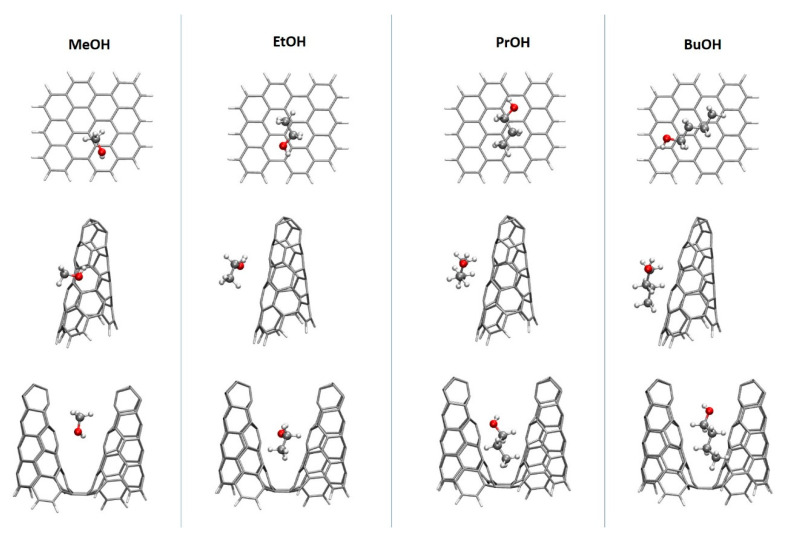
The most stable complexes of studied alcohols on: a graphene sheet and on SWCNH (ωB97X-D/6-31G(d,p)).

This is why in DFT calculations we assume the leading role of dispersion interactions in studied systems. Figure 6 collects the equilibrated configurations of n-alcohols on a graphene plane and on SWCNH. For this case we show the results for single and double—cone system.

The exemplary complexes have been selected from the four of the initial geometries as the low-energy complexes in which alcohol is adsorbed in a way that minimizes the influence of the edge of the material and in a comparable mode for all four alcohols investigated (namely in similar position with respect to the material). Due to the character of the materials and the lack of the heteroatoms in the models, the preferred interactions are of dispersion nature (see the results collected in Table 1).

Therefore, in general, the alcohols tend to arrange the carbon chains in parallel to the material surface, gaining the more stabilization the longer alcohol chain is. The only exception from this rule is methanol adsorption on graphene sheet, where hydrogen from the hydroxyl group prefers to be pointing to the material plane directly in all of the analyzed initial structures. Such a behavior can be reasoned by the additional exploitation of the –OH...π contact in the case when the alcohol chain is too short to benefit much from the pure dispersion. In the case of double cones with the frozen distance between the tips of the cones, the alcohol enters the space between the walls, benefiting from the dispersion stabilization from both sides. Thus, as could be expected, the supermolecular attraction energy is smaller for the single cones than for planar graphene sheets, since the curvature of the nanocone walls decreases the optimal stacking of the carbonaceous systems. However, the addition of the second cone to the model provides another stabilizing fragment of a material, leading to the increase of the interaction energy with respect to the single nanocone adsorbent. The alcohol adsorption on the double cone is even more advantageous than on the graphene plane. As for experimental systems also the linear correlation between energy and molecular diameter (polarizability) is observed.

**Figure 7:**
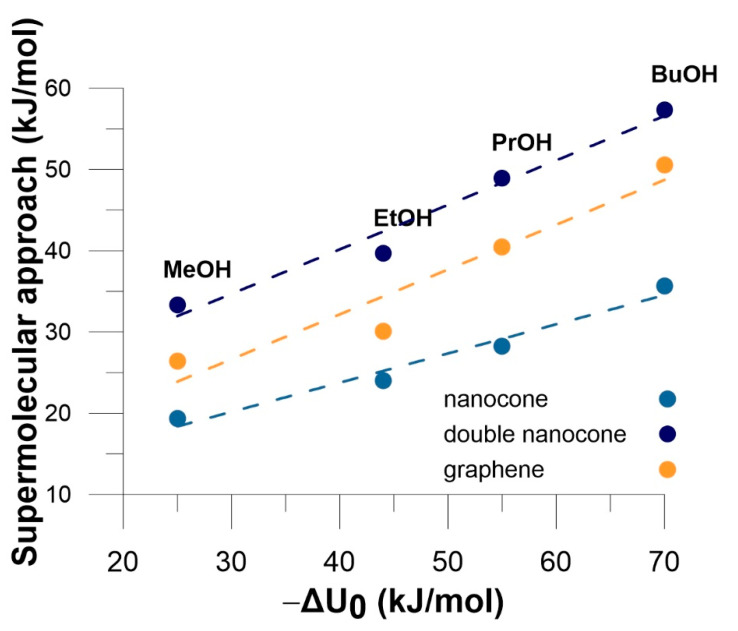
The relation between the energies calculated using DFT and the values of experimental enthalpy of adsorption at zero coverage.

The data collected on Figure 7 additionally shows that the values of solid—fluid interaction energies calculated from the DFT are in good agreement with experimental data. In fact, double cone model leads to more realistic energy values. However, we can observe that even for this model, the energy of interaction for EtOH, PrOH and BuOH is still a little too small. It means that the potential energy of dispersion interactions with nanohorn should be increased, probably by introducing additional nanocones to the system. This, however, will leads to drastic elongation of calculation time.

## 4. Conclusions

Chromatographic measurements of n-alcohols adsorption on SWCNHs at low coverages are reported for the first time. Adsorption of studied n-alcohols increases with the chain length. Obtained isosteric enthalpy of adsorption values also increase in the same order. The values of enthalpy at zero—coverage suggest that the adsorption centers of SWCNH are a little less homogeneous than observed on the surface of a graphite. On the other hand, the correlation of experimentally determined adsorption enthalpy with the energy values calculated using the DFT suggests that the number of heteroatoms on the surfaces of SWCNH is negligibly small. Proposed double cone model leads to more realistic DFT energy values, however it needs a small improvement. Increasing the alcohol alkyl chain leads to preferring of the orientation with OH group to the cone ends. In this way alcohol molecules are entrapped in potential energy minima by alkyl chains interactions, and the ratio of dispersion to electrostatic interactions energy in studied systems increases (Table 1). This is very promising for the future application of SWCNHs in catalysis, and the results will be reported.

## Figures and Tables

**Table 1:** The components of Equation (2) for studied systems.

Graphene	Interaction Energy and Its Components (SAPT0/aug-cc-pVDZ), (kJ/mol)						
	Elst	Exch	Ind	Disp	Total SAPT0	SCS-SAPT0	Disp/Elst
MeOH	-	-	-	-	-	-	-
1	−15.03	28.47	−5.48	−38.56	−30.65	−22.06	2.56
2	−10.89	26.17	−5.07	−38.10	−27.88	−19.38	3.50
3	−13.44	27.17	−5.28	−37.60	−29.14	−20.77	2.80
4	−10.97	26.38	−5.07	−38.18	−27.88	−19.38	3.48
EtOH	-	-	-	-	-	-	-
1	−11.30	28.76	−2.93	−43.29	−28.76	−19.18	3.83
2	−11.47	28.13	−5.28	−41.45	−30.20	−20.81	3.62
3	−17.83	42.41	−5.36	−56.81	−37.64	−25.04	3.19
4	−17.83	42.41	−5.36	−56.84	−37.64	−25.04	3.18
PrOH	-	-	-	-	-	-	-
1	−18.25	39.56	−4.02	−58.82	−41.53	−28.47	3.22
2	−17.00	38.43	−3.85	−57.11	−39.48	−26.84	3.36
3	−22.02	54.09	−6.28	−75.57	−49.78	−33.03	3.43
4	−18.25	39.48	−3.98	−57.53	−40.28	−27.55	3.15
BuOH	-	-	-	-	-	-	-
1	−19.80	44.92	−4.73	−58.70	−38.31	−25.37	2.96
2	−19.43	43.79	−4.14	−64.81	−44.59	−30.23	3.34
3	−25.87	63.97	−7.24	−88.47	−57.61	−38.06	3.42
4	−24.41	62.59	−6.82	−91.86	−60.54	−40.15	3.76

## Data Availability

The data presented in this study are available on request from the corresponding author.
